# Short-Chain Fatty-Acid-Producing Micro-Organisms Regulate the Pancreatic FFA2-Akt/PI3K Signaling Pathway in a Diabetic Rat Model Affected by Pumpkin Oligosaccharides

**DOI:** 10.3390/foods12193559

**Published:** 2023-09-25

**Authors:** Guimei Liu, Bin Yu, Jianpeng Li, Zheng Zhang, Haiteng Tao, Haibo Zhao, Yanmin Lu, Chao Yuan, Quanhong Li, Bo Cui

**Affiliations:** 1 State Key Laboratory of Biobased Material and Green Papermaking, School of Food Science and Engineering, Qilu University of Technology, Shandong Academy of Sciences, Jinan 250353, China; mml1011@163.com (G.L.); yubin@qlu.edu.cn (B.Y.); jianpenglee@qlu.edu.cn (J.L.); zhengzhang324@163.com (Z.Z.); taohaiteng@163.com (H.T.); haibozhao@qlu.edu.cn (H.Z.); yanminlu2008@aliyun.com (Y.L.); yuanchao@qlu.edu.cn (C.Y.); 2 National Engineering Research Center for Fruit and Vegetable Processing, College of Food Science and Nutritional Engineering, China Agricultural University, Beijing 100083, China; quanhong_li@hotmail.com

**Keywords:** pumpkin oligosaccharides, gut microbiota, SCFAs, FFA2-Akt/PI3K, type 2 diabetes

## Abstract

Herein, we applied the Illumina MiSeq pyrosequencing platform to amplify the V3–V4 hypervariable regions of the 16 S rRNA gene of the gut microbiota (GM) and a gas chromatograph–mass spectrometer to detect the metabolites after supplementation with pumpkin oligosaccharides (POSs) to determine the metabolic markers and mechanisms in rats with type 2 diabetes (T2D). The POSs alleviated glucolipid metabolism by decreasing the serum low-density lipoprotein (LDL), total cholesterol (TC), and glucose levels. These responses were supported by a shift in the gut microbiota, especially in the butyric-acid-producing communities. Meanwhile, elevated total short-chain fatty acid (SCFA), isovaleric acid, and butyric acid levels were observed after supplementation with POSs. Additionally, this work demonstrated that supplementation with POSs could reduce TNF-α and IL-6 secretion via the FFA2-Akt/PI3K pathway in the pancreas. These results suggested that POSs alleviated T2D by changing the SCFA-producing gut microbiota and SCFA receptor pathways.

## 1. Introduction

The polysaccharides extracted from pumpkin (Cucurbita moschata, Duch) have attracted increasing attention in recent years because of their antidiabetic, antihyperlipidemic, and oxidation-resistance effects, their functional values, and their variety of nutrients [1,2]. However, our previous study showed that alcohol-precipitation-isolated macromolecular polysaccharides have more carbohydrates in the supernatant [2]. We hypothesized that the bioactivity of pumpkin polysaccharides might be limited by their complex structure and high molecular weight. Therefore, ultrafiltration (3000 Da) and nanofiltration (300 Da) were performed to prepare and characterize the oligosaccharides extracted from fresh pumpkins using the membrane filter method [3]. Due to their biological activity and physicochemical properties, oligosaccharides are good for human health and can be applied in the food industry and biotechnology [4]. However, it is unknown whether pumpkin oligosaccharides (POSs) could be used as a prebiotic to attenuate type 2 diabetes mellitus (T2DM) or obesity by changing the GM. 

As a metabolic disorder, T2DM is associated with metabolic disturbances with regard to proteins, fats, and carbohydrates, and it induces low-grade systemic inflammation and peripheral insulin resistance in response to a high-fat diet (HFD) [5,6,7]. Additionally, a systematic metagenome-wide association study reported that T2DM is associated with the gut microbiota (GM) [8]. Moreover, diabetes drugs alleviate glucose homeostasis [9] and gut hormones [10], and they are closely associated with the GM of patients with T2DM. These results suggest that the GM is no longer a ‘forgotten organ’. Indeed, the 1–2 kg of gut micro-organisms containing >150-fold more genes than the human genome itself have attracted the attention of researchers from the fields of microbiology, physiology, and gastroenterology [11,12]. Polysaccharides are reported to decrease serum lipopolysaccharide, TNF-α, and IL-6 by influencing the short-chain fatty acid (SCFA)-producing GM [13]. SCFAs are intermediate products of the fermentation of dietary fiber by gut micro-organisms, which partially convert sugars, proteins, and peptides into SCFAs, such as butyric acid, propionic acid, and acetic acid [14]. Based on these results, we can use SCFA markers to study the mechanisms by which dietary-fiber-producing gut microbes improve the outcomes of obesity and metabolic diseases, including T2DM. 

Dietary supplements or diabetes drugs alleviate T2DM associated with SCFA-producing gut micro-organisms. However, the effects of POSs on the GM in T2DM remain unclear, and no direct evidence regarding the relationship between SCFAs, the gut microbiota, and POSs is available. Thus, to compare the changes in the GM, gut metabolic profile, and SCFAs induced by POSs in HFD/STZ-induced T2DM rats, we used next-generation sequencing (NGS) via the Illumina MiSeq pyrosequencing platform. This systematic analysis might provide a novel understanding of the interactions between micro-organisms and the host, the key biomarkers, and the underlying mechanisms induced by POSs in metabolic diseases. 

## 2. Methods and Materials

### 2.1. Oligosaccharide Preparation 

As described in our previous study [3], we prepared and characterized oligosaccharides extracted from fresh pumpkins, excluding the seeds and rind (Cucurbita moschata, Duch). After crushing the fresh pumpkin and mixing it with three volumes of water, a 3 h incubation in a water bath at 90 °C with a rotating speed of 170 rpm was carried out. Then, after cooling, a 20 min centrifugation at 10,000 rpm was carried out. To concentrate the supernatant, rotary evaporation (four times) and 80% ethanol (three times) were applied. Then, a 20 min centrifugation at 8000 rpm was carried out to remove the polysaccharides. To separate the supernatant, the membrane filter method was performed using ultrafiltration (3000 Da) followed by nanofiltration (300 Da). Finally, after concentration and freeze-drying, the collected fractions were prepared and used in animal experiments. 

### 2.2. Animal Experimental Design

The animal experiments were carried out according to the guidelines of China’s Institutional Animal Care and Use Committee, and all the procedures performed were consistent with the European Community guidelines (2010/63/EU) for the care and use of experimental animals. In order to eliminate the effect of sex on feed intake and the results of glucolipid metabolism, we conducted the animal experiments using adult male Wistar rats (body weight of 200 g, *n* = 50). All the animals were kept in a controlled SPF environment at 22 ± 1 °C, at 42–47% humidity, and under a 12/12 h light/dark cycle (lights on 06:00–18:00). Additionally, the animals had free access to food and water during this study. After one week of acclimatization, the rats received a high-fat diet (HFD) composed of 66.5% pulverized standard rat pellet, 1% sodium cholate, 2.5% cholesterol, 20% sucrose, and 10% lard for four weeks. After the rats had fasted overnight, 30 mg/kg STZ (Sigma) was given to the HFD-fed rats via intravenous (i.v.) injection. A vehicle control buffer was given to the normal control rats. Two weeks after the STZ injection, we examined the fasting blood glucose (FBG) concentration, and the rats with a FBG > 11.1 mmol/L (*n* = 30) were selected for further experiments. 

Next, the T2D rats were divided into three groups: (1) a nontreated group (NC, *n* = 10): rats fed with HFD during the experiment; (2) an HFD + POS group (*n* = 10): HFD-fed rats treated intragastrically with 500 mg/kg pumpkin oligosaccharides once daily according to a toxicology test; and (3) an HFD + metformin (MET) group (*n* = 10): HFD-fed rats treated intragastrically with 200 mg/kg metformin once daily [15]. 

### 2.3. Sample Collection

We collected blood samples via ocular sampling once the rats were anesthetized. Afterward, they were beheaded and fresh fecal samples (rectum content) and pancreatic tissue were collected at the end of the experiment. As previously described [16], to collect the serum samples at the end of the trial, 15 min centrifugation at 1100× *g* was conducted after 2 h of clotting of the blood samples at room temperature (RT). Then, rapid freezing of the pancreatic tissue and fecal samples was carried out using liquid nitrogen. The blood glucose (GLU), HDL-C, triglyceride (TG), total cholesterol (TC), and LDL-C contents of the serum were examined by the Health Science Center of Peking University as previously described [17]. The short-chain fatty acid receptor 2 (FFA2), interleukin-6 (IL-6), pancreatic and duodenal homeobox 1 (PDX1), 3-phosphoinositide-dependent protein kinase 1 (PDK1), tumor necrosis factor-α (TNF-α), phosphoinosmde-3-kinase (PI3K), and protein kinase B (Akt) expression in the pancreatic tissue was detected. The primer sequences for FFA2, IL-6, PDX1, PDK1, PI3K, and Akt are listed in Supplementary Table S1. Additionally, the gut microbiota and the profiles of the metabolites in the colon contents were also determined. The data were from all the rats, excluding the dead one and when there was a failure to obtain samples. 

### 2.4. Gut Microbiota Detected Using Illumina MiSeq Pyrosequencing Platform

Based on a previous description [18], a TIANamp Stool DNA Kit (TIAGEN, Beijing, China, DP328) was employed to prepare the genomic DNA from the colon contents. Nanodrop (Thermo Scientific, Waltham, MA, USA) and agarose gel electrophoresis were used to determine the quantity and quality of the DNA samples. To obtain the V3–V4 hypervariable regions of the 16S rRNA gene in the microbiota via Polymerase Chain Reaction (PCR), we employed primers (F 5-ACTCCTACGGGAGGCAGCA-3 and R 5-GGACTACHVGGGTWTCTAAT-3) according to a previous study [19]. The PCR mixture (25 μL) contained 0.25 μL of Q5 DNA polymerase, 8.75 μL of ddH_2_O, 2 μL of DNA template, 1 μL of reverse primer (10 µM), 1 μL of forward primer (10 µM), 2μL of dNTP (2.5 mM), 5 μL of 5 × GC buffer, and 5 μL of 5 × reaction buffer. The PCR procedure was as follows: 98 °C, 2 min for initial denaturation; 98 °C, 15 s for denaturation; 55 °C, 30 s for annealing; 72 °C, 30 s for extension, repeated for 25 cycles; and 72 °C, 5 min for the final extension. After purification of the PCR product using gel electrophoresis followed by AxyPrep DNA Gel Extraction (Axygen, Corning, NY, USA, AP-GX-500), a TruSeq Nano DNA LT Library Prep Kit (Illumina, San Diego, CA, USA, FC-121-4001) was used to construct the DNA library. After testing the results using an Agilent High Sensitivity DNA Kit (Agilent, Santa Clara, CA, USA, 5067-4626), Illumina MiSeq was used to sequence the samples.

### 2.5. Metabolic Profile Quantified via Gas Chromatography–Mass Spectrometry (GC–MS) 

GC–MS was used to detect the profiles of the metabolites and SCFAs. Briefly, 100 mg fecal samples were placed in 5 mL centrifuge tubes with 500 μL of ddH_2_O and mixed at 4 °C for 90 s. After the addition of heptadecanoic acid (60 μL, 0.2 mg/mL in methanol), 2-chloro-lphenylalanine (60 μL, 0.2 mg/mL in methanol), and −20 °C pre-cooled methanol (1000 μL), the samples were vortexed for 30 s. Then, the samples were treated for 10 min with ultrasound at RT and cooled on ice for 30 min. Next, centrifugation was carried out for 10 min at 12,000 rpm and 4 °C. Then, the supernatant (1.2 mL) was blow-dried using vacuum concentration. Then, methoxyamine pyridine solution (60 μL, 15 mg/mL) was added, and the reaction was carried out for 120 min at 37 °C. Next, the mixture was reacted for 90 min at 37 °C with 60 μL of BSTFA reagent containing 1% TMCS. Next, after the 10 min centrifugation at 12,000 rpm and 4 °C, the supernatant was transferred and stored for analysis.

For the separation of the derivatives, an HP-5MS capillary GC column (with 0.25 μm film thickness, 30 m × 250 μm i.d., and 5% phenyl/95% methylpolysiloxane) was applied. For the injection of the samples (1 µL), an autosampler was used in split mode and a 20:1 split ratio. The injection, interface, and ion source temperatures were 280, 150, and 230 °C, respectively. The temperature was increased as follows: initial temperature: 60 °C for 2 min; 10 °C/min until 300 °C; and 300 °C for 5 min. To determine the results of the mass spectrometry, the full-scan method was used from 35 to 750 (m/z).

### 2.6. Quantification of the SCFA Profiles

We mixed standard concentrations of caproic acid, isovaleric acid, valeric acid, isobutyric acid, butyric acid, propionic acid, and acetic acid with ethyl acetate to 200, 100, 50, 20, 10, 5, 1, and 0.1 μg/mL, respectively. After resuspension of the colon contents (100 mg) with 0.5% phosphoric acid (1 mL) in 2 mL sterile tubes, 10 min centrifugation at 17,949× *g* was conducted to collect the supernatant (800 μL). Then, after a 2 min oscillation of the supernatant with ethyl acetate (800 μL), the samples were centrifuged again (17,949 g/10 min). After mixing 600 μL of the organic phase with 500 μM 4-methylpentanoic acid, each sample was collected into a gas-phase flask to analyze the SCFA contents.

An Agilent 7890A/5975C system equipped with a DB-WAX MS silica capillary column (30 m × 0.25 mm × 0.25 μm) was used for the GC–MS analysis. After 1 min of maintenance of the oven temperature at 90 °C, the temperature was raised to 120 °C (10 °C/min). The oven temperature was then increased to 150 °C (5 °C/min) and then to 250 °C (25 °C/min). The injection port temperature was set at 250 °C. During each run, 1 μL of the prepared sample was injected using helium as the carrier gas at a 1.0 mL/min flow rate with a split ratio of 10:1.

### 2.7. Gene Expression Detection 

The gene expression was assayed using real-time quantitative PCR (RT-qPCR) [20]. Briefly, after extracting the total RNA from the pancreas tissues with TRIzol (Invitrogen, Carlsbad, CA, USA), the quality and quantity were assessed using a biophotometer (Eppendorf, Eppendorf, Germany). The 18S and 28S rRNA subunits were evaluated using agarose gel electrophoresis, and a reverse reaction system was prepared with 10 U of ribonuclease inhibitor, 0.7 nmol/L of oligo d (T), 2.5 U of AMV, 1 mmol/L of dNTP, 1 μL of RT buffer, 5 mmol/L of MgCl_2_, and 500 ng of total RNA. For the PCR, an SYBR green master mix (TaKaRa Biotechnology, Dalian, China) was employed in ABI7500 [20]. GAPDH expression was used to normalize the target gene expression. The comparative cycle threshold (CT) method (2-ΔΔCT) was used to quantify the mRNA expression according to the method described by Livak and Schmittgen [21].

### 2.8. Statistical Analysis

One-way variance analysis in SAS (version 9.4) (SAS INSTITUTE INC, Raleigh, NC, USA)was carried out to analyze the effects of POSs on the SCFAs, gene expression, and biochemical parameters. Data are listed as the square means ± SD. Means with different letters are significantly different, *p* < 0.05. QIIME (Quantitative Insights Into Microbial Ecology, v1.8.0) was used to analyze the Illumina MiSeq sequences. Based on the linear discriminant analysis (LDA), which was conducted using the Wilcoxon rank sum and Kruskal–Wallis tests (*p* < 0.05), we employed mothur software (https://mothur.org/, accessed on 2 September 2023) (University of Michigan, Ann Arbor, MI, USA ) and identified the key communities for separating the different treatment groups (Shanghai Personal Biotechnology Co., Ltd., Shanghai, China). Spearman analysis (*p* < 0.05) was carried out to evaluate the connections of the SCFAs to key GM communities. 

## 3. Results and Discussion

### 3.1. Physiological and Biochemical Properties 

To investigate the hypoglycemic and hypolipidemic effects of POSs, STZ-induced T2D rats were continued on an HFD. The concentration of serum GLU significantly decreased (*p* = 0.03) with POS supplementation for four weeks compared to the NC group (Table 1). In addition, POSs significantly decreased the concentration of TC in the blood compared to the control treatment without an HFD (*p* = 0.03). We also detected a remarkable reduction in the TG concentration (*p* = 0.01) when POSs and MET were administrated intragastrically for four weeks. The changes in the lipid metabolism parameters were similar to those in a study in which T2DM rats were treated with a hypoglycemic diguanide drug, presenting decreased serum TG, TC, and LDL-c levels [22]. Although an evident reduction (*p* = 0.06) in the serum LDL-c content was detected under POS supplementation, no differences were detected in the MET group. The serum HDL-C did not differ (*p* = 0.69) between the control, POS, and metformin groups. There is no direct evidence regarding the effects of POSs on T2D in terms of lipid metabolism and glucose attenuation. However, as a hypoglycemic diguanide drug for oral usage, MET is significantly connected to glycemic control [23], lipid metabolism [22], and anti-inflammatory functions [24]. POSs, which have a similar function in the biochemical index, might be a potential component of food for improving metabolic diseases, and this should be further investigated in the food industry.

### 3.2. Gut Microbiota Shifts and Profile

**Figure 1:**
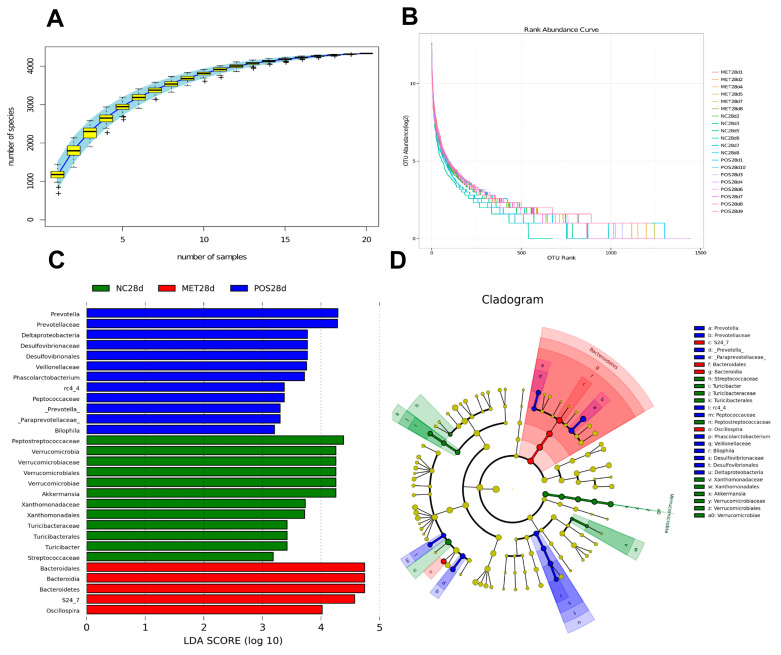
The richness of the gut microbiota: (**A**) rank abundance curve. The cumulative species curvs reflects the influence of sample number on species diversity. “+” is abnormal value; and (**B**) species accumulation curves under POS treatment. Significantly different species based on the LDA score (**C**) and LEfSe taxonomy cladogram (**D**) identified using the nonparametric factorial Kruskal–Wallis rank sum test at a significance level of 0.05.

Prebiotics [25], probiotics [26], and dietary style, such as a Mediterranean diet [27], can all regulate the balance of the intestinal microflora and induce a shift in the gut microbiota toward a healthy intestinal environment [28]. In the present study, the Illumina MiSeq sequencing system was used to examine the effects of POSs as a prebiotic on the gut microbiome profile of T2D rats. The high-quality sequences were delineated into 94,542 OTUs with a similarity cutoff of 97% (Figure 1A). Through a rarefaction analysis, we captured most of the gut microbial diversity (Figure 1B). The nonparametric factorial Kruskal–Wallis rank sum test was used to detect significantly different species between the treatments at a significance level of 0.05 (Figure 1C,D, Supplementary Table S2). In the group receiving POS supplementation, significantly different communities (*p* < 0.05) were identified, belonging to Bacteroidetes, Proteobacteria, and Firmicutes (Supplementary Table S2). The LEfSe taxonomy cladogram was used to show the relative abundance of the significantly different species (*p* < 0.05) (Figure 1D). The LDA score of >2 obtained for this group was considered a higher relative abundance than that obtained for the other two groups (*p* < 0.05), and the POS group showed the selective enrichment of Prevotella, Paraprevotellaceae, Prevotellaceae, Phascolarctobacterium, rc4_4, Deltaproteobacteria, and Bilophila (LAD scores: 3.30, 3.30, 4.28, 3.72. 3.37, 3.77, and 3.21, respectively) (Figure 1C, Supplementary Table S2). Salgaço states that the composition of the intestinal microbiota and the induction of low-grade inflammation play significant roles in T2DM [29]. In the present work, the relative abundance of S24_7 and Oscillospira, belonging to the Bacteroidetes and Firmicutes phyla, was increased in the rats treated with MET (Supplementary Table S2). This result is consistent with research in which the enrichment of Bacteroidetes participated significantly in the metabolism of complex molecules, such as xylan, pectin, and cellulose, into simple molecules, which can be used as energy sources during MET treatment [30,31]. Although there was a similar shift found in the POS supplementation and diabetic drug groups, the key enteric micro-organisms differed between these groups. Our results regarding POS supplementation are supported by research that reported a positive relationship between T2DM and Enterobacteriaceae and Deltaproteobacteria [32]. We also detected a change in the enrichment of Bilophila after POS supplementation, which was supported by other results concerning the increasing relative abundance of Bilophila and Turicibacter being promoted by whole-grain barley and malt-treated barley [33].

### 3.3. Gut Metabolic Profiling via GC–MS

**Figure 2:**
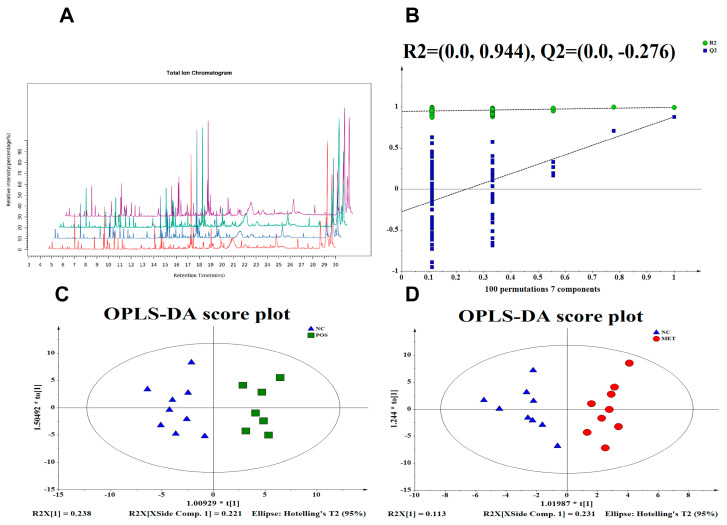
Effects on the gut metabolic structure determined using the orthogonal partial least squares discriminant analysis method: (**A**) representative GC−MS total ion chromatograms (TIC); (**B**) OPLS−DA permutation test between the POS (Green), MET (Red), and NC (Blue); and OPLS−DA score plot between (**C**) POS and NC and (**D**) MET and NC.

Next, we compared and overlayed the total ion chromatography (TIC) spectra of the quality control samples. There was an overlap between the retention time and response intensity of the quality control samples, which suggested that this model had low variation, good stability, and robustness, and a low instrumental error (Figure 2A). The overall distribution changes in the GM and the predictive ability and fitness of our model were assessed using orthogonal partial least squares discriminant analysis (OPLS-DA) (Figure 2B). Differences were observed between the POS and MET treatments and the gut metabolic profiles of the NC group, indicating that the gut metabolites were different between the POS and control groups (Figure 2C). The differences identified in the metabolite contents are supported by a shift in the gut microbiota and the glucolipid metabolism parameters, which demonstrated the antidiabetic effects of POS supplementation in T2DM rats. Based on the above results, we speculated that the antidiabetic mechanisms of POSs are related to structural changes in metabolites in the gut (Figure 2D).

We identified the key metabolites in the different treatments based on the VIP (variable importance in the projection) value. For the biomarkers differentially expressed in the varied treatments, metabolites with *p* < 0.05 and VIP > 1 were defined as significantly differential, while 0.05 < *p* < 0.1 and VIP > 1 were considered differential [34]. Our study identified 31 potential gut metabolites (Table 2) when supplementing with POSs compared to the NC group. Among these metabolites, butyric acid, valeric acid, and isovaleric acid were upregulated with a 1.38, 2.02, and 1.1 Log2 fold change (FC). Our results are supported by Sanchez et al., who stated that plant polysaccharides and oligosaccharides resist enzymatic and chemical digestion until they reach the large intestine, where they are fermented into SCFAs by the gut microbiota [35]. Similar results were also obtained by other researchers. Studies have indicated that the fermentation of complex polysaccharides produces acetate, propionate, and butyrate in the gut [36]. 

### 3.4. SCFA Concentration in the Gut 

To investigate whether SCFAs were key mediators of POSs within the GM in the improvement of T2DM, the SCFAs in the colon contents were analyzed. The concentrations of caproic acid, isovaleric acid, valeric acid, isobutyric acid, propionic acid, butyric acid, and acetic acid are shown in Table 3. Previous research has reported that bacterial fermentation of resistant starch and fiber in the colon mainly produces butyrate, propionate, and acetate [37]. In this work, the butyric acid concentration exhibited a significant increase (*p* < 0.01) after POS treatment in the HFD T2D rats, consistent with the metabolite detection results. We also observed a significant increase (*p* < 0.01) in the acetic acid concentration under POS supplementation compared to the control group. Valeric acid, isobutyric acid, and propionic acid also increased. An evident elevation in the total SFCAs (*p* = 0.03) was detected after POS treatment in the T2DM rats. The results of the gut metabolic analysis indicate that SCFAs, especially butyric and isovaleric acids, are the key biomarkers of the antidiabetic effect of pumpkin oligosaccharides in T2DM rats.

### 3.5. Spearman Correlation of Key Species and SCFAs 

**Figure 3:**
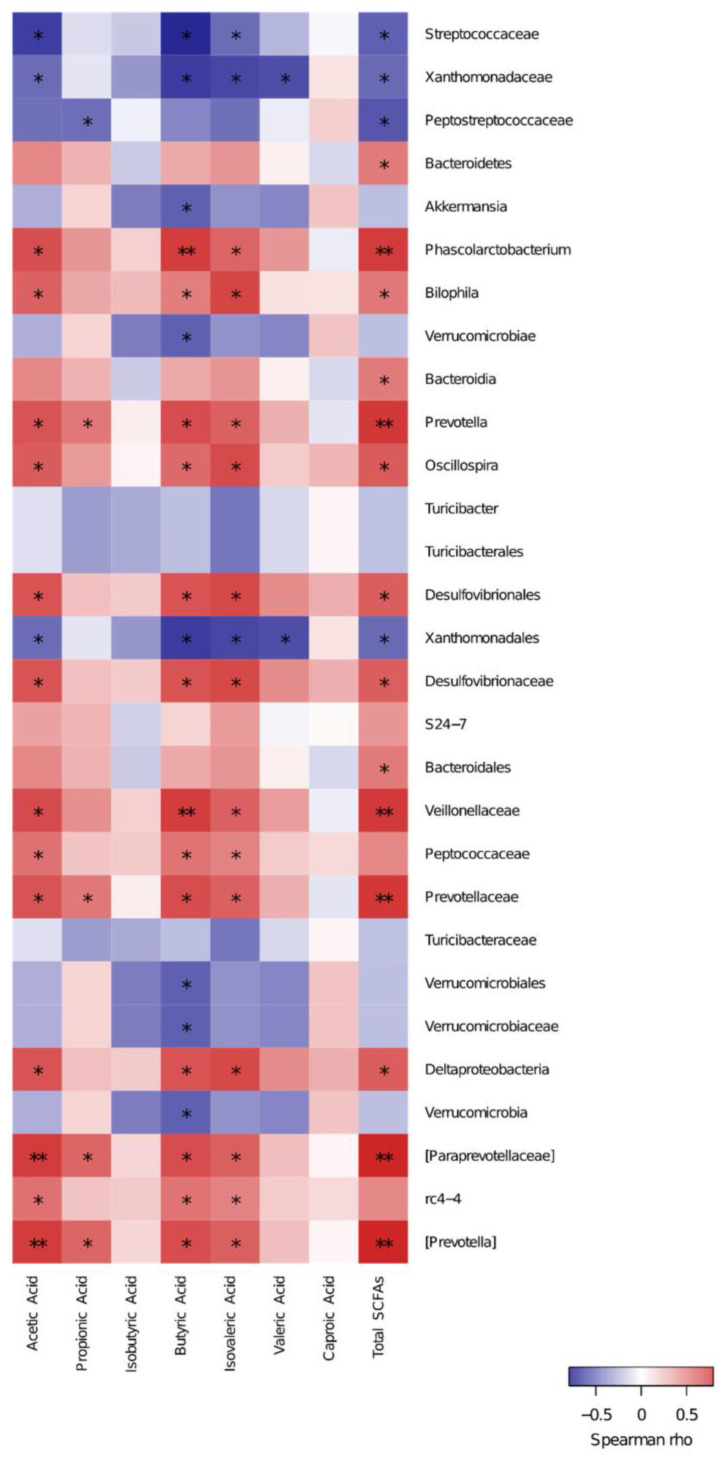
Spearman’s correlations of key species and SCFAs; ** *p* < 0.01 and * *p* < 0.05.

Many studies have shown that microbes are significantly connected to the metabolism of SCFAs [38,39]. The metabolism of complex carbohydrates changes the diversity of the gut microbiota and produces SCFAs, which are associated with gut microbiota diversity [40]. To determine whether SCFAs are key metabolic biomarkers related to changes in enteric micro-organisms, Spearman’s rank correlation coefficient was applied to analyze the relationship using the key communities obtained from the LEfSe results by pooling all the groups together (Figure 3, Supplementary Table S3). Phascolarctobacterium, Bilophila, Prevotella, Oscillospira, Desulfovibrionales, Desulfovibrionaceae, Veillonellaceae, Peptococcaceae, Prevotellaceae, Deltaproteobacteria, and rc4-4 showed a positive (*p* < 0.05) correlation with butyric acid (Spearman coefficients: 0.70, 0.47, 0.65, 0.54, 0.62, 0.62, 0.70, 0.50, 0.65, 0.62, and 0.50, respectively). These key species presented the same trend for the acetic and isovaleric acids. Meanwhile, Streptococcaceae, Xanthomonadaceae, and Xanthomonadales presented negative correlations (*p* < 0.05) with isovaleric acid, butyric acid, and acetic acid. The enrichments of Prevotellaceae and Paraprevotellaceae were correlated with propionic acid with coefficients of 0.49 and 0.55. NGS and metabolomics profiling were used to evaluate the effects of POSs on the gut microflora and metabolites in T2DM. We found that butyric acid was strongly correlated with the key communities under POS treatment, consistent with similar experiments on the cross-feeding between Akkermansia muciniphila and butyrate-producing bacteria promoting butyrate production [41,42]. Additionally, the Mediterranean diet, which is largely plant-based, increases the availability of substrates that can be fermented by the gut microbiota, leading to SCFA formation [43]. Elevated hemoglobin A1c and GLP-1 levels and an increased abundance of butyrate-producing bacteria were found in SCFA-supplemented T2DM patients [44]. The significance of the gut microbiota in the metabolic regulation of SCFAs has been demonstrated by many studies. However, the SCFAs that participate in the regulation of glucolipid metabolism and the mechanisms underlying this regulation in T2DM have not been fully elucidated. 

### 3.6. SCFA-Related Gene Expression in Pancreatic Tissues

SCFAs play their roles by binding to FFA2 and FFA3, which are considered G-protein-coupled receptors and act as free fatty acid receptors [45]. There is a large amount of direct evidence concerning the functions of FFA2, FFA3, and SCFAs. For example, FFA2 knockout induces colitis, gout, and arthritis by disrupting the immune system in mice [46]. Meanwhile, acetate supplementation via drinking water mitigates this response in germ-free mice [47], and FFA2-knockout mice remain refractory to acetate treatment [48]. Because relatively high expressions of FFA2 and FFA3 are present in the β cells of islets in humans and rodents [49], we detected the mRNA levels of the FFA2 gene in pancreatic tissues(Table 4). We found that the FFA2 expression was dramatically enhanced by POSs at the transcriptional level (*p* < 0.01). In the present study, the pancreatic tissues from POS-treated rats showed dramatically reduced TNF-α and IL-6 expression at the transcriptional level (*p* < 0.01). This result is supported by another study using chitosan oligosaccharides, indicating decreased TNF-α and resistin levels, as well as increased adiponectin, during treatment in T2DM patients [50]. TNF-α and IL-6 are significantly involved in diabetes, as they induce pancreatic β cell apoptosis, especially in Streptozotocin-induced dysfunction of the pancreas [2]. We hypothesized that SCFAs alleviate inflammation through the FFA pathway in T2DM rats, which is supported by findings that acetate inhibited LPS-induced TNFα secretion via FFA receptor pathway activation in mice and human mononuclear cells [51]. Simultaneously, we detected the increased expression of the PI3K and AKT genes in the pancreas when the rats were supplemented with POSs, which played a pivotal role in improving the immunologic function by activating inflammatory mediators and inflammatory cell recruitment [52]. Based on the above research and the expression of the relative genes, there is a suggestion that the FFA2-Akt/PI3K pathway is the route through which SCFAs regulate inflammatory cytokines under POS treatment in T2DM rats. Therefore, future research will be required to identify and study indigestible carbohydrates in the relationship between the FFA2-Akt/PI3K signaling pathway and inflammatory cytokines at the protein level in vitro and in vivo.

## 4. Conclusions

Our present work demonstrated the beneficial effects of POSs in an STZ HFD-induced T2DM rat model. The administration of POSs decreased the serum GLU, TC, and TG levels; decreased the pancreatic IL-6 and TNF-α gene expression; and increased the gut SCFAs and the pancreatic mRNA levels of Akt and PI3K. Simultaneously, we detected the key species through microbiome analysis using 16s rRNA sequencing and the key metabolites using GC–MS after POS treatment in T2DM rats. The Spearman correlation analysis revealed that POSs fermented by enteric micro-organisms induced the generation of SCFAs. SCFAs circulated to peripheral tissues play an important role by binding to the FFA2 receptor, which might be the mechanism underlying the improvements in inflammation in the pancreas. The changes in Akt/PI3K supported this notion. The selective enrichment of the SCFA-producing communities and the FFA2-Akt/PI3K pathway indicated that POS treatment has a potential therapeutic effect on T2DM by improving glucolipid metabolism. Further research could focus on the deep macromolecular mechanisms underlying the effects of macromolecular polysaccharides and oligosaccharides on biomarkers from the gut microbiota.

## Figures and Tables

**Table 1:** Changes in Glu, TC, TG, LDL-C, and HDL-C after the indicated treatments.

	NC	POS	MET	*p* Value
Glu (mmol/L)	26.66 ± 5.24 ^a^	17.62 ± 3.92 ^b^	21.68 ± 3.50 ^b^	0.03
TC (mmol/L)	5.23 ± 0.35 ^a^	3.13 ± 0.40 ^b^	3.03 ± 0.33 ^b^	0.03
TG (mmol/L)	1.78 ± 0.21 ^a^	1.00 ± 0.09 ^b^	1.05 ± 0.23 ^b^	0.01
LDL-C (mmol/L)	2.59 ± 0.32 ^a^	1.31 ± 0.28 ^b^	2.59 ± 0.56 ^a^	0.06
HDL-C (mmol/L)	1.23 ± 0.14	1.47 ± 0.09	1.09 ± 0.10	0.69

Data are listed as the square means ± SD. Means with different letters are significantly different, *p* < 0.05. NC: negative control, *n* = 8; POS: pumpkin oligosaccharides group, *n* = 8, MET: metformin treatment group, *n* = 9.

**Table 2:** The key metabolites determined via GC–MS.

Metabolites	VIP		*p* Value		Log_2_fc_	
	POS	MET	POS	MET	POS	MET
4-hydroxy-proline	1.10	0.82	0.00	0.38	−2.61	−0.73
9-(Z)-hexadecenoic acid	1.73	0.60	0.00	0.48	−1.21	−0.17
9-(Z)-octadecenoic acid	1.44	0.52	0.01	0.33	−1.08	−0.21
Alanine	1.49	0.70	0.01	0.09	−1.00	−0.31
Aspartic acid	1.51	1.56	0.01	0.11	1.16	1.18
Butyric acid	1.33	1.76	0.01	0.01	1.38	0.96
Cholesterol	1.15	0.62	0.03	0.54	−0.65	−0.19
Docosahexaenoic acid	1.15	0.66	0.01	0.79	−1.54	−0.38
Fructose	1.34	1.53	0.02	0.08	−1.34	−0.72
Fucose	1.22	1.10	0.03	0.29	−0.93	−0.49
Glucaric acid	1.01	0.02	0.01	0.38	−1.45	−0.02
Glutamine	0.49	0.07	0.53	0.60	−0.35	0.03
Glycerol	1.4	1.74	0.01	0.05	−1.44	−0.86
Glycerol-3-phosphate	1.14	1.10	0.00	0.09	−1.05	−0.49
Hexadecanoic acid	1.38	0.35	0.02	0.54	−0.30	0.09
Homoserine	1.42	0.61	0.02	0.43	−0.94	−0.24
Isoleucine	0.71	0.33	0.04	0.06	−0.34	−0.11
Isomaltose	1.29	1.64	0.00	0.02	−1.66	−1.07
Isovaleric acid	1.38	0.44	0.02	0.38	1.10	−0.18
Lactic acid	0.69	1.02	0.03	0.03	−2.99	−2.37
Lactose	1.15	0.85	0.03	0.48	−0.86	−0.32
Leucine	0.75	1.56	0.02	0.01	−1.18	−1.52
Malonic acid	1.34	1.54	0.00	0.08	−1.51	−0.81
Mannose	1.15	1.01	0.03	0.60	−1.64	−0.60
Methionine	0.58	0.71	0.40	0.38	−0.27	−0.19
Nicotinic acid	1.17	0.61	0.06	0.93	−0.86	−0.18
Ornithine	1.12	0.44	0.06	0.66	−0.61	−0.15
Oxalic acid	1.7	3.09	0.00	0.00	−1.13	−1.86
Proline	0.63	0.54	0.02	0.66	−0.35	−0.19
Propionic acid	1.01	1.00	0.01	0.29	1.16	0.60
Pyruvic acid	1.04	0.94	0.03	0.16	−1.17	−0.61
Serine	1.06	1.14	0.14	0.25	−0.43	−0.31
Sucrose	1.39	1.04	0.01	0.16	−1.05	−0.41
Threonic acid	1.39	1.57	0.01	0.08	−1.69	−0.96
Threonine	0.83	0.86	0.34	0.54	−0.39	−0.26
Trans-sinapinic acid	1.16	1.09	0.00	0.05	−1.35	−0.70
Valeric acid	1.25	1.17	0.00	0.08	2.02	0.85
Valine	0.65	0.33	0.04	0.03	−0.27	0.22
Xylitol	1.24	1.49	0.03	0.09	−1.12	−0.70

Differences in metabolites represented by VIP (variable importance in the projection) > 1, *p* value < 0.05, or FC (fold change) ≥ 1.5 or ≤ 0.667.

**Table 3:** The concentrations of SCFAs determined via GC–MS.

μg/g	NC	POS	MET	*p* Value
Acetic acid	1505.2 ± 82.92 ^b^	2208.6 ± 121.56 ^a^	1718.1 ± 108.93 ^b^	<0.01
Propionic acid	1244.2 ± 201.89	1468.2 ± 291.65	1641.5 ± 378.17	0.68
Isobutyric acid	60.25 ± 8.24	90.68 ± 15.58	82.38 ± 16.68	0.34
Butyric acid	352.86 ± 34.96 ^c^	899.42 ± 61.75 ^a^	589.92 ± 89.32 ^b^	<0.01
Isovaleric acid	46.88 ± 4.88 ^b^	97.52 ± 12.60 ^a^	82.11 ± 6.11 ^a^	0.06
Valeric acid	90.86 ± 20.00	143.72 ± 18.63	125.36 ± 11.96	0.13
Caproic acid	32.85 ± 0.18	33.38 ± 0.51	32.78 ± 0.27	0.50
Total SCFAs	3333.1 ± 312.19 ^b^	4941.5 ± 320.58 ^a^	4272.2 ± 507.34 ^ab^	0.03

Data are listed as the square means ± SD. Means with different letters are significantly different, *p* < 0.05. NC: negative control, *n* = 8; POS: pumpkin oligosaccharides group, *n* = 8, MET: metformin treatment group, *n* = 9.

**Table 4:** Effects of POSs on gene expression in the pancreas in T2D rats.

	NC	POS	MET	*p* Value
FFA2	1.00 ± 0.00 ^c^	4.97 ± 0.23 ^a^	2.73 ± 0.18 ^b^	<0.01
IL-6	1.00 ± 0.00 ^a^	0.57 ± 0.02 ^b^	0.17 ± 0.0.03 ^c^	<0.01
TNF-α	1.00 ± 0.00 ^a^	0.61 ± 0.03 ^b^	0.21 ± 0.03 ^c^	<0.01
PDK1	1.00 ± 0.00	1.13 ± 0.06	1.09 ± 0.04	0.32
PDX1	1.00 ± 0.00	1.18 ± 0.05	1.21 ± 0.04	0.58
PI3K	1.00 ± 0.00 ^b^	2.41 ± 0.12 ^a^	1.97 ± 0.16 ^a^	<0.01
Akt	1.00 ± 0.00 ^b^	2.62 ± 0.15 ^a^	2.51 ± 0.17 ^a^	<0.01

Data are listed as the square means ± SD. Means with different letters are significantly different, *p* < 0.05. NC: negative control, *n* = 8; POS: pumpkin oligosaccharides group, *n* = 8, MET: metformin treatment group, *n* = 9.

## Data Availability

The datasets generated for this study are available on request to the corresponding author.

## Supplementary Materials

The following supporting information can be downloaded at: https://www.mdpi.com/article/10.3390/foods12193559/s1, Table S1: Gene-specific primers used for mRNA expression measurements; Table S2: The significant differently species determined based on the LEfSe method using the nonparametric factorial Kruskal-Wallis rank sum test; Table S3: Relationship between key communities of gut microbiota and SCFAs by pumpkin oligosaccharide or metformin treatment.
