# Supplementary material for: Short-Chain Fatty-Acid-Producing Micro-Organisms Regulate the Pancreatic FFA2-Akt/PI3K Signaling Pathway in a Diabetic Rat Model Affected by Pumpkin Oligosaccharides
Source: Foods. 2023 Sep 25;12(19):3559. doi: 10.3390/foods12193559 (PMC10572525; doi:10.3390/foods12193559)
Supplement: Supplementary file 1 [file foods-12-03559-s001.zip › foods-2618462-supplementary.pdf]

Table S1 Gene-specific primers used for mRNA expression measurements.

Gene	GenBank Accession No.	Primers position	Primers sequences(5’→3’)
FFA2	NM_005306.3	Forward	TGCTACTACTCCGCAATCCC
		Reverse	CTGTGCCCCTTCTGTTTGAC
IL-6	NM_012589.2	Forward	GCCACTGCCTTCCCTACTTC
		Reverse	TCTGACAGTGCATCATCGCT
TNF-α	L00981.1	Forward	TCTGGAGAGCAAACACGGAC
		Reverse	ATGGGAGTAGGAATGGCCCT
PDX1	NM_022852.4	Forward	ACAAATACATCTCCCGGCCT
		Reverse	CTTCATGCGACGGTTTTGGA
PDK1	XM_032914423	Forward	CCCCGATTCAAGTTCACGTC
		Reverse	TCTGTCGATCTTCCTCAGCG
PI3K	NM_0130051	Forward	GAGGATTTGCCCCACCATGA
		Reverse	ACGCAATGTTTGACTTCGCC
Akt	NM_0033230	Forward	GAGAACCGTGTCCTGCAGAA
		Reverse	GTTCTCCAGCTTGAGGTCCC
GAPDH	NM_017008.4	Forward	GGTGGACCTCATGGCCTACA
		Reverse	CTCTCTTGCTCTCAGTATCCTTGCT

Table S2 The significant differently species determined based on the LEfSe method using the nonparametric factorial Kruskal-Wallis rank sum test.

Treat	Taxon	Relative abundance(log10)	LDA Score	P Value
NC	Firmicutes.Bacilli.Lactobacillales.Streptococcaceae	2.87	3.18	0.01
	Firmicutes.Bacilli.Turicibacterales	3.85	3.42	0.01
	Firmicutes.Bacilli.Turicibacterales.Turicibacteraceae	3.85	3.42	0.01
	Firmicutes.Bacilli.Turicibacterales.Turicibacteraceae.Turicibacter	3.85	3.42	0.01
	Firmicutes.Clostridia.Clostridiales.Peptostreptococcaceae	4.96	4.38	0.03
	Proteobacteria.Gammaproteobacteria.Xanthomonadales	1.65	3.72	0.01
	Proteobacteria.Gammaproteobacteria.Xanthomonadales.Xanthomonadaceae	1.65	3.73	0.01
	Verrucomicrobia	4.66	4.26	0.02
	Verrucomicrobia.Verrucomicrobiae	4.66	4.26	0.02
	Verrucomicrobia.Verrucomicrobiae.Verrucomicrobiales	4.66	4.26	0.02
	Verrucomicrobia.Verrucomicrobiae.Verrucomicrobiales.Verrucomicrobiaceae	4.66	4.26	0.02
	Verrucomicrobia.Verrucomicrobiae.Verrucomicrobiales.Verrucomicrobiaceae.Akkermansia	4.66	4.26	0.02
MET	Bacteroidetes	5.18	4.74	0.02
	Bacteroidetes.Bacteroidia	5.18	4.74	0.02
	Bacteroidetes.Bacteroidia.Bacteroidales	5.18	4.74	0.02
	Bacteroidetes.Bacteroidia.Bacteroidales.S24_7	5.02	4.57	0.03
	Firmicutes.Clostridia.Clostridiales.Ruminococcaceae.Oscillospira	4.40	4.02	<0.01
POS	Bacteria.Bacteroidetes.Bacteroidia.Bacteroidales._Paraprevotellaceae_._Prevotella_	3.64	3.30	0.01
	Bacteroidetes.Bacteroidia.Bacteroidales._Paraprevotellaceae_	3.64	3.30	0.01
	Bacteroidetes.Bacteroidia.Bacteroidales.Prevotellaceae	4.68	4.28	0.01
	Bacteroidetes.Bacteroidia.Bacteroidales.Prevotellaceae.Prevotella	4.68	4.29	0.01

Firmicutes.Clostridia.Clostridiales.Peptococcaceae	3.63	3.37	0.01
Firmicutes.Clostridia.Clostridiales.Peptococcaceae.rc4_4	3.63	3.37	0.01
Firmicutes.Clostridia.Clostridiales.Veillonellaceae	4.08	3.76	<0.01
Firmicutes.Clostridia.Clostridiales.Veillonellaceae.Phascolarctobacterium	4.05	3.72	<0.01
Proteobacteria.Deltaproteobacteria	4.06	3.77	0.01
Proteobacteria.Deltaproteobacteria.Desulfovibrionales	4.06	3.77	0.01
Proteobacteria.Deltaproteobacteria.Desulfovibrionales.Desulfovibrionaceae	4.06	3.77	0.01
Proteobacteria.Deltaproteobacteria.Desulfovibrionales.Desulfovibrionaceae.Bilophila	3.40	3.21	0.01

NC: control group; MET: metformin treatment group; POS: pumpkin oligosaccharide treatment group.

Table S3 Relationship between key communities of gut microbiota and SCFAs by pumpkin oligosaccharide or metformin treatment

Community	Acetic.Acid	Propionic.Acid	Isobutyric.Acid	Butyric.Acid	Isovaleric.Acid	Valeric.Acid	Caproic.Acid	Total.SCFAs
	Spearman' correlation							
Streptococcaceae	-0.60*	-0.10	-0.17	-0.67*	-0.46*	-0.23	-0.02	-0.49*
Xanthomonadaceae	-0.45*	-0.08	-0.33	-0.60*	-0.57*	-0.55*	0.10	-0.46*
Peptostreptococcaceae	-0.44	-0.45*	-0.04	-0.37	-0.44	-0.06	0.18	-0.52*
Bacteroidetes	0.43	0.28	-0.16	0.31	0.39	0.05	-0.12	0.48*
Akkermansia	-0.25	0.16	-0.41	-0.49*	-0.33	-0.38	0.21	-0.20
Phascolarctobacterium	0.64*	0.39	0.17	0.70**	0.56*	0.38	-0.06	0.71**
Bilophila	0.57*	0.32	0.24	0.47*	0.67**	0.11	0.10	0.49*
Verrucomicrobiae	-0.25	0.16	-0.41	-0.49*	-0.33	-0.38	0.21	-0.20
Bacteroidia	0.43	0.28	-0.16	0.31	0.39	0.05	-0.12	0.48*
Prevotella	0.62*	0.49*	0.06	0.65*	0.57*	0.29	-0.08	0.72**
Oscillospira	0.59*	0.37	0.04	0.54*	0.66*	0.19	0.27	0.59*
Turicibacter	-0.10	-0.30	-0.26	-0.20	-0.43	-0.12	0.03	-0.19
Turicibacterales	-0.10	-0.30	-0.26	-0.20	-0.43	-0.12	0.03	-0.19
Desulfovibrionales	0.62*	0.23	0.19	0.62*	0.66*	0.42	0.29	0.59*
Xanthomonadales	-0.45*	-0.08	-0.33	-0.60*	-0.57*	-0.55*	0.10	-0.46*
Desulfovibrionaceae	0.62*	0.23	0.19	0.62*	0.66*	0.42	0.29	0.59*

S24-7	0.34	0.26	-0.14	0.15	0.36	-0.03	0.02	0.38
Bacteroidales	0.43	0.28	-0.16	0.31	0.39	0.05	-0.12	0.48
Veillonellaceae	0.65*	0.41	0.17	0.70**	0.57*	0.36	-0.05	0.72**
Peptococcaceae	0.52*	0.22	0.19	0.50*	0.45*	0.19	0.14	0.44
Prevotellaceae	0.62*	0.49*	0.06	0.65*	0.57*	0.29	-0.08	0.72**
Turicibacteraceae	-0.10	-0.30	-0.26	-0.20	-0.43	-0.12	0.03	-0.19
Verrucomicrobiales	-0.25	0.16	-0.41	-0.49*	-0.33	-0.38	0.21	-0.20
Verrucomicrobiaceae	-0.25	0.16	-0.41	-0.49*	-0.33	-0.38	0.21	-0.20
Deltaproteobacteria	0.62*	0.23	0.19	0.62*	0.66*	0.42	0.29	0.59*
Verrucomicrobia	-0.25	0.16	-0.41	-0.49*	-0.33	-0.38	0.21	-0.20
[Paraprevotellaceae]	0.71**	0.55*	0.15	0.65*	0.58*	0.23	0.03	0.79**
rc4-4	0.52*	0.22	0.19	0.50*	0.45*	0.19	0.14	0.44
[Prevotella]	0.71**	0.55*	0.15	0.65*	0.58*	0.23	0.03	0.79**
